# Editorial: Drug resistance in breast cancer – mechanisms and approaches to overcome chemoresistance

**DOI:** 10.3389/fonc.2022.1080684

**Published:** 2023-01-04

**Authors:** Dayanidhi Raman, Anca Maria Cimpean, Maria Rosaria De Miglio

**Affiliations:** ^1^ Department of Cell and Cancer Biology, University of Toledo, Toledo OH, United States; ^2^ Department of Microscopic Morphology/Histology, Victor Babes University of Medicine and Pharmacy, Timisoara, Romania; ^3^ Angiogenesis Research Center Timisoara, Victor Babes University of Medicine and Pharmacy, Timisoara, Romania; ^4^ Center of Expertise for Rare Vascular Disease in Children, Emergency Hospital for Children Louis Turcanu, Timisoara, Romania; ^5^ Department of Medicine, Surgery and Pharmacy, University of Sassari, Sassari, Italy

**Keywords:** breast cancer, drug resistance, stem-like cells, non-coding RNAs, triple- negative breast cancer

According to the GLOBOCAN program 2020, breast cancer (BC) had the highest incidence among women worldwide, with an estimated 2.3 million new cases, corresponding to 11.7% of all cancer cases. It is the fifth leading cause of cancer mortality in the world, with 685.000 deaths (1). Conventional therapeutic approaches for BC include radical surgical resection, radiotherapy, chemotherapy, and endocrine therapies, which induce cancer cell death. Recently, immunotherapy and targeted therapies have altered the prognoses of patients with BC, improving their life quality and survival (2), (3). Despite significant improvement in the outcomes of BC patients, many of them present intrinsic drug resistance, while others are initially drug-sensitive but acquire resistance to anticancer drugs, and frequently multidrug resistance, leading to recurrence and/or metastasis (4–6). Furthermore, growing evidence revealed that patients with the same BC molecular subtype can have different responses to treatment, strongly supporting the high BC heterogeneity. Currently, drug resistance is a major reason for poor prognosis, reducing survival in BC patients (7). If drug resistance could be defeated the impact on BC patient survival would be significant.

Multiple mechanisms linked to drug resistance have been explained in BC treatment, including somatic mutations or epigenetic changes within drug targets, cancer cell heterogeneity, cancer stem cells, cancer-associated macrophages and immune cells modulation, metabolic reprogramming, and interactions among cancer cells and tumor microenvironment (8). Substantial evidence linked therapy resistance to aberrations in mi*RNA* (microRNA, small, single-stranded, non-coding RNA molecules containing 21 to 23 nucleotides) expression levels, which in turn cause dysregulation of gene expression (9). Alyami argued that the main rationale for targeting miRNA is how their involvement in miRNA-mRNA complex networks can manipulate cell apoptosis, cell cycle, epithelial-mesenchymal transition (EMT), and drug resistance, making it an exclusive therapeutic target.

Tian et al. summarized the mechanisms of ncRNAs (non-coding RNA) in chemotherapeutic, endocrine, and targeted drug resistance in BC patients. They described ncRNAs as a target gene of drugs influencing its effects by acting as ce*RNAs* (competing endogenous RNA that regulate other RNA transcripts by competing for shared mi*RNAs*). This mechanism modulated neoplastic cell sensitivity and drug resistance, regulating cancer apoptosis and cell cycle transfer, and inducing modulation of various signaling pathways. Moreover, Tian et al. suggested that targeting nc*RNA*s could be a novel strategy for achieving improved treatment outcomes for BC patients.

Recently, the PARP inhibitor Olaparib was approved for the treatment of triple- negative breast cancer (TNBC) with BRCA mutations (10), although differences in the sensitivity of individual patients and resistance to Olaparib have been shown (11). Interesting results of Zhao et al. showed that the sensitivity of TNBC cells to Olaparib can be increased by inducing overexpression in neoplastic cells miR-27-3p, which targets sPSEN-1, the catalytic subunit of γ-secretase, and blocks the activation of the Notch pathway *via* the inhibition of the cleavage of the Notch protein.

The failure of current therapies and the consequent high mortality in BC patients is greatly ascribed to the therapy-resistant cancer stem cells (CSCs) present in the bulk of the tumor. Often, CSCs increase the drug efflux transporters and mostly stay in the non-dividing cell-cycle phase (G0) to escape conventional therapeutics, and the induced residual disease is responsible for tumor progression (12). As summarized by Saha et al., the recognition of deregulated miRNAs/ncRNAs/mRNAs signatures in CSCs and their crosstalk with multiple pathways uncovered potential therapeutic targets in drug-resistant BC. Moreover, therapies that can induce alternative mechanisms of cell death, such as ferroptosis, pyroptosis, immunotherapy, drugs targeting CSC metabolism, and nanoparticle therapy, are the upcoming approaches to target the CSCs and overcome drug resistance.

The leading cause of BC death is disease progression due to metastases. Because of this challenge, the identification of unambiguous molecular biomarkers to predict the disease response is needed. Biomarkers are independent and measurable assessments of biological states or diseases that can be critical for the appropriate management of BC during treatment (Saha et al.).

Li et al. identified STAT5a as a key promoter of doxorubicin (DOX)-resistance in BC, inducing overexpression of the multidrug resistance protein ABCB1. Moreover, the use of the STAT5 inhibitor Pimozide, initially approved by the FDA as a psychotropic diseases drug, significantly increased BC cells’ sensitivity to DOX both *in vitro* and *in vivo*. STAT5a could be a promising therapeutic target for the treatment of chemoresistant BC, with Pimozide being a likely candidate to reduce chemoresistance. Zhang et al. provided evidence that complement component C7 expression (a 93-kDa serum glycoprotein encoded by the *C7* gene, one of the main components of Membrane Attacking Complex-MAC) was an independent poor prognostic factor in triple- negative and luminal B BC subtypes. Furthermore, patients with high C7 expression were insensitive to taxane-anthracycline (TE)-based chemotherapy. These findings highlighted the importance of C7 in BC progression and set a foundation to help clinicians improve the identification of patients for TE chemotherapy by determining C7 expression in the era of precision medicine.

Extracellular adenosine triphosphate (eATP) is abundant in the tumor microenvironment. eATP is highly toxic to BC cells despite being easily degraded by eATPases. Chemotherapy induces further increases in eATP through P2RX channels of cancer cells. Interesting results obtained by Manouchehri et al., demonstrated that eATP is toxic to several TNBC cell lines, and that the purinergic channels P2RX4 and P2RX7 are necessary for this effect. Chemotherapy exposure induced the release of eATP from TNBC cell lines, and inhibitors of eATP metabolism augmented chemotherapy-induced loss of TNBC cell viability.

Cyclin-dependent kinases 4 and 6 (CDK4 and CDK6) are enzymes important in cell division. For ER-positive and HER2-negative metastatic BC, endocrine therapy (ET) combined with CDK4/6 inhibitors is the gold standard for first- and second-line treatment (13). Nevertheless, resistance to this combination therapy frequently develops in this BC subtype. Kim et al. developed a prediction model based on five clinical and preclinical factors: (i) primary resistance to adjuvant ET; (ii) liver metastasis; (iii) initial CA-15-3 elevation; (iv) weak ER expression, and (v) BRCA2 mutations. Based on this prediction model, patients with ER-positive and HER2-negative metastatic BC may benefit from an initial examination to help identify subgroups at risk of developing primary resistance to the first-line treatment of Palbociclib with Letrozole. In ER-positive and HER2-negative metastatic BC patients, this prediction model-based first evaluation may aid oncologists in the early identification of a group at high risk of acquiring primary resistance to combined therapy with Palbociclib and Letrozole. This group of individuals has a significant propensity for acquiring medication resistance, thus additional treatment options should be considered.

Furthermore, Mao et al. showed that PR-negativity was a significant prognostic factor for total pathological complete response (pCR) or breast pCR rate in HER2 positive BC subtype treated with pyrotinib-containing neoadjuvant therapy.

More frequently, anticancer drug candidates result in translational failures in clinical trials and the main reason for this failure can be attributed to non-accurate preclinical models. Law et al. summarized that to ensure drug efficacy and its mechanism of action has clinical translatability, the complexity of the tumor microenvironment needs to be appropriately modeled. 3D culture models are emerging as powerful research tools that recapitulate *in vivo* characteristics. Technological advancements in this field show promising applications in improving drug discovery, pre-clinical validation, and precision medicine.

## Conclusions

**Figure 1:**
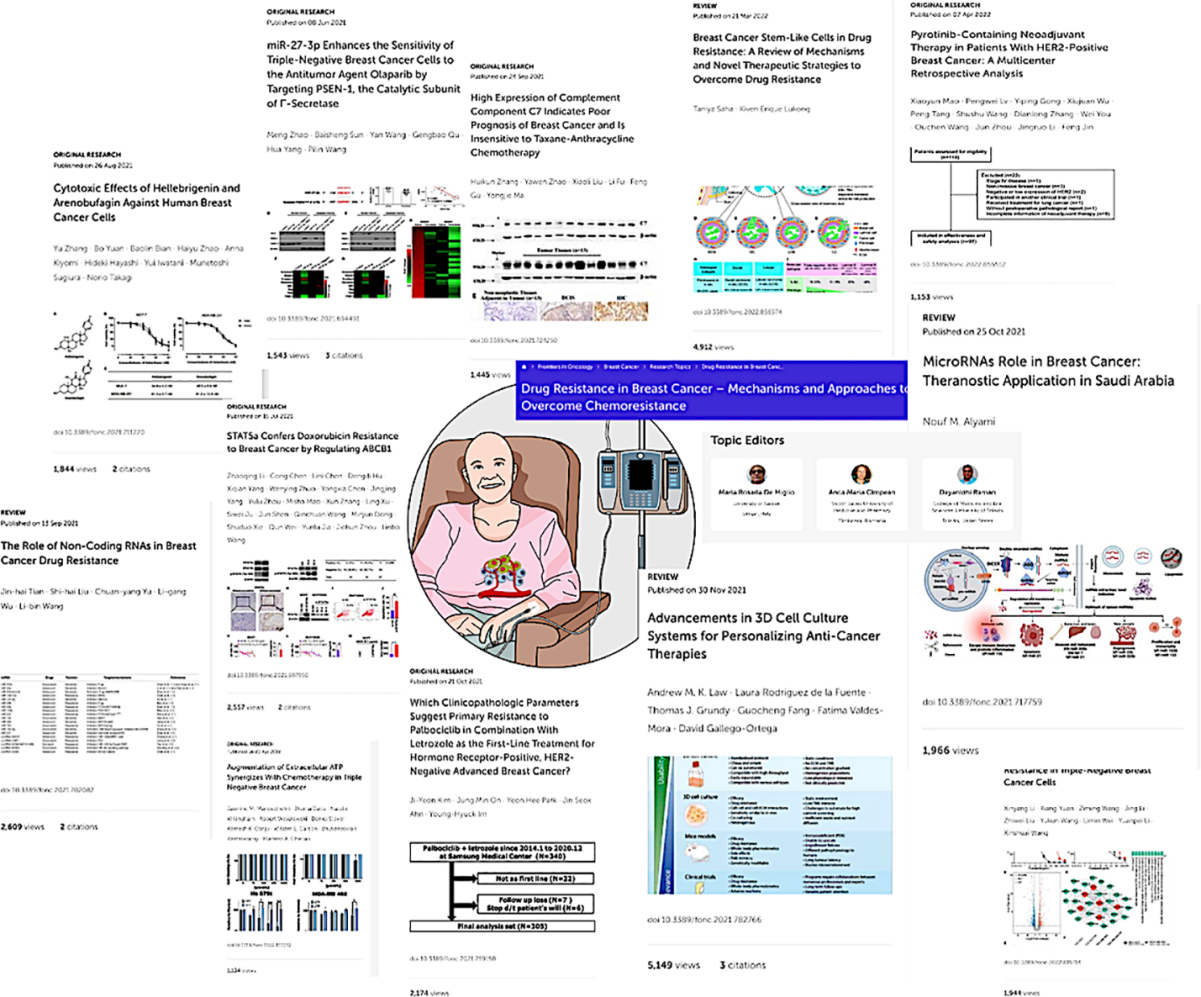
Overview illustration of the Research Topic Special Issue. Centered on patients and respecting them, authors, Frontiers in Oncology Editorial Board, guest editors, and reviewers worked together to build a successful and useful issue. All papers submitted to this issue were valuable but 14 of them (inserted in the picture) were the most valuable for patients, doctors, and researchers.

Breast cancer remains one of the most challenging diseases, with unexpected behavior even after several years of remission. Breast cancer molecular classification represented a big step in the elucidation of this malignant heterogeneity, but it seems that it is not enough to explain some hidden sides of the disease, especially the development of therapy resistance. The papers published in the Research Topic -Drug Resistance in Breast Cancer – Mechanisms and Approaches to Overcome Chemoresistance in Frontiers in Oncology (**Figure 1**) suggested that molecular classification needs to be improved by adding new criteria derived from more accurate experimental and clinical data. Also, this Research Topic highlighted the invasion ability and metastasis of breast cancer stem cells and their response and resistance to current therapies. This Research Topic contains a well-balanced proportion of valuable clinical and experimental studies in the field of breast cancer, which are extremely useful for both clinicians and academic researchers.

## Author contributions

All authors listed have made a substantial, direct, and intellectual contribution to the work and approved it for publication.

## References

[1] Sung H Ferlay J Siegel RL Laversanne M Soerjomataram I Jemal A. Global cancer statistics 2020: GLOBOCAN estimates of incidence and mortality worldwide for 36 cancers in 185 countries. CA Cancer J Clin (2021) 71:209–49. doi: 10.3322/CAAC.21660 33538338

[2] Esteva FJ Hubbard-Lucey VM Tang J Pusztai L. Immunotherapy and targeted therapy combinations in metastatic breast cancer. Lancet Oncol (2019) 20:e175–86. doi: 10.1016/S1470-2045(19)30026-9 30842061

[3] Masoud V Pagès G. Targeted therapies in breast cancer: New challenges to fight against resistance. World J Clin Oncol (2017) 8:120–34. doi: 10.5306/WJCO.V8.I2.120 PMC5385433 28439493

[4] Abad E Graifer D Lyakhovich A. DNA Damage response and resistance of cancer stem cells. Cancer Lett (2020) 474:106–17. doi: 10.1016/J.CANLET.2020.01.008 31968219

[5] Marquette C. Chemotherapy-resistant metastatic breast cancer. Curr Treat Options Oncol (2012) 13:263–75. doi: 10.1007/s11864-012-0184-6 22528367

[6] Brown KA Andreopoulou E Andreopoulou P. TOUCH MEDICAL MEDIA endocrine therapy-related endocrinopathies-biology, prevalence, and implications for the management of breast cancer. Rev Breast Cancer J Publ Date (2020) 6:17–22. doi: 10.17925/OHR.2020.16.1.17 PMC8034602 33841882

[7] Longley DB Johnston PG. Molecular mechanisms of drug resistance. J Pathol J Pathol (2005) 205:275–92. doi: 10.1002/path.1706 15641020

[8] Hu W Tan C He Y Zhang G Xu Y Tang J. OncoTargets and therapy dovepress functional miRNAs in breast cancer drug resistance. Onco Targets Ther (2018) 11:1529–41. doi: 10.2147/OTT.S152462 PMC5865556 29593419

[9] Mulrane L Mcgee SF Gallagher WM O’connor DP. miRNA dysregulation in breast cancer. (2013). doi: 10.1158/0008-5472.CAN-13-1841 24204025

[10] Eikesdal HP Yndestad S Elzawahry A Llop-Guevara A Gilje B Blix ES. Olaparib monotherapy as primary treatment in unselected triple negative breast cancer. Ann Oncol (2021) 32:240–9. doi: 10.1016/J.ANNONC.2020.11.009 33242536

[11] Moustafa D Abd Elwahed MR Elsaid HH Parvin JD. Modulation of early mitotic inhibitor 1 (EMI1) depletion on the sensitivity of PARP inhibitors in BRCA1 mutated triple-negative breast cancer cells. PloS One (2021) 16:e0235025. doi: 10.1371/JOURNAL.PONE.0235025 33412559 PMC7790533

[12] Wang J Liu X Jiang Z Li L Cui Z Gao Y. A novel method to limit breast cancer stem cells in states of quiescence, proliferation or differentiation: Use of gel stress in combination with stem cell growth factors. Oncol Lett (2016) 12:1355–60. doi: 10.3892/OL.2016.4757/HTML PMC4950051 27446437

[13] Cardoso F Paluch-Shimon S Senkus E Curigliano G Aapro MS André F. 5th ESO-ESMO international consensus guidelines for advanced breast cancer (ABC 5). Ann Oncol (2020) 31:1623–49. doi: 10.1016/J.ANNONC.2020.09.010 PMC7510449 32979513

